# Inconsistent condom use for prevention of HIV/STIs among street-based transgender sex workers in Lahore, Pakistan: socio-ecological analysis based on a qualitative study

**DOI:** 10.1186/s12889-023-15550-w

**Published:** 2023-04-03

**Authors:** Shermeen Bano, Rahla Rahat, Florian Fischer

**Affiliations:** 1 Department of Sociology, Forman Christian College (A Chartered University), Lahore, Pakistan; 2 Institute of Social and Cultural Studies, University of the Punjab, Lahore, Pakistan; 3 Institute of Public Health, Charité – Universitätsmedizin Berlin, Berlin, Germany

**Keywords:** HIV, Sex work, Inconsistent condom use, HIV risks, Barriers

## Abstract

**Background:**

A large amount of new HIV infections worldwide is observed amongst key populations which include e.g., commercial sex workers or transgender people and their respective sexual partners. Therefore, this study examined the multi-level context of inconsistent condom use (ICU) in sexual interactions of transgender street-based workers (KSWs) with commercial and non-commercial sexual partners in Lahore.

**Methods:**

We conducted 20 in-depth interviews with street-based KSWs to investigate challenges to consistent condom utilization with sexual partners. The qualitative data was analyzed using reflexive thematic analysis to produce an initial set of codes and identify broader themes through a recursive exploration of the text.

**Results:**

Within a socio-ecological analysis we identified factors influencing ICU among KSWs at three levels. At the individual level, we identified knowledge and awareness, age, pleasure and pain, and mental health issues impacting on ICU. perceived characteristics of sexual partners, dynamics of cruising spots and places of sexual interactions, competition in sex trade, violence and lack of safety nets in street-based sex work, and condom use with lovers were factors associated with ICU. Risk factors at community level were changing urban geography of sex work, discrimination, harassment and regular evictions, networks with non-governmental organizations and the influence of gurus and Dera culture.

**Conclusions:**

Until now, HIV prevention efforts in Pakistan have primarily focused on HIV risk factors at the level of individual behaviors within specific networks of target populations. However, our study points towards both the effectiveness and the urgency of interventions that address macro-level risk factors specific to key populations in Pakistan, in addition to behavioral interventions.

## Introduction

Inconsistent condom use (ICU) among commercial sex workers, is one of the leading sources of spread of Sexually Transmitted Infections (STIs) including HIV around the world [1]. Due to widespread discrimination and socio-economic exclusion of gender and sexual minorities, transgender sex workers are disproportionately affected by STIs and HIV [2] and face multiple barriers in access and utilization of condoms during commercial and non-commercial sexual interactions [3].

The term “transgender people” is used by the World Health Organization (WHO) to refer to all individuals whose internal sense of gender (gender identity) and expression diverge from the social expectations of their biological sex at birth [4]. In Pakistan, transgender people are locally referred to as *Khwaja Sira.* The Khawaja Sira community in Pakistan includes a variety of gender nonconforming people [5]. High rates of HIV among Khwaja Sira sex workers (KSWs) have been noted, and it was estimated that one out of every five KSWs in Pakistan would be living with HIV by the end of 2020 [6]. In addition, hepatitis B, human papillomavirus (HPV), and genital herpes are key sexually transmitted diseases (STDs) affecting Khwaja Sira populations that can be prevented through condom use [7, 8]. Condom use, however, is alarmingly low among KSWs in Pakistan. According to the most recent nation-wide HIV surveillance data, consistent condom use among KSWs was low; 13.1% with clients and 6.7% with non-paid partners. In addition, KSWs with less than 10 years of education were twice as more likely to not use condoms consistently than those with more than 10 years of education [9]. In another study, only 45% of KSWs respondents were found to be using condoms consistently with their clients [1].

Socio-cultural and economic contexts are paramount in shaping sexual behaviors including condom use [10]. Multi-level factors of ICU among commercial sex workers have been identified. Age [11], level of education, duration of sex work, total number of clients, knowledge of HIV and STIs, affordability and accessibility of condoms [12], and perception of risk [13] are important contributing factors of ICU at individual level [1]. At structural level, location of sex work [14–16], policing of sex workers and criminalization of sex work [17, 18] have been found to impact ICU. In addition, research has found that sex without condoms is higher-priced due to client demand [19] and condom use is identified as damaging to business among commercial sex workers [20]. For instance, many customers of KSWs in Pakistan lack adequate knowledge of safe sex practices and are either suspicious of condoms [21] or prefer sex without condoms for seeking sexual pleasure [22]. As a result, studies indicate that concerns for preferences of clients discourages KSWs from using condoms [23].

This paper examines ICU in KSWs within the context of street-based sex work in Lahore by applying the Modified Social Ecological Model (MSEM) [24]. The MSEM examines HIV risk at five levels, ranging from the micro-level of the individual to meso-levels of network and community, and ultimately to the macro-level defined by public policy and the stage of HIV epidemic. Currently there is a dearth of research on ICU within the particular social, physical, and occupational contexts of street-based KSWs in Pakistan. Applying a social-ecological framework to ICU among KSWs can help to determine the way in which the multi-level socio-structural injustices and oppression experienced by street-based KSWs contribute to and reinforce barriers to condom use during commercial and non-commercial sexual interactions.

### Khawaja Sira sex work in Pakistan

South Asia has a rich history of gender ambiguity whose contours have shifted with changing landscapes of authority in the region. In precolonial India, members of the Khwaja Sira community were viewed as pious individuals due to the ambiguity of their sexual corporeality. They upheld prestigious ranks in state and society, and distinguished themselves from impoverished and socially marginalized *hijras* who cross-dressed, engaged in sex work and resided in impoverished neighborhoods. However, colonization of India by the British resulted in the establishment of policies that led to criminalization and regulation of transgender populations [25]. The transgender bodies were pathologized as “diseased” and Khwaja Siras lost their high ranks within the society and the newly established government [26]. As a result of the loss of livelihood, Khwaja Siras found support in the hijra community which led to the assimilation of these two populations, their identities and practices and to the marginalized position they are in today [27]. Recently, in an attempt to reclaim their historical respect and status, the term *Khwaja Sira* has been adopted and encouraged for all transgender people by transgender activists in place of the derogatory term *hijra* [26]. This has been corresponded by granting of citizenship privileges and rights to Khwaja Siras through the Pakistani Supreme Court’s rulings in 2012 and the passing the Transgender Protection Bill in 2018. Despite these political changes in Pakistan, economic and social deprivation of Khawaja Siras continues as a result of which many members of this community remain dependent on sex work for survival [27].

Sex work is not a recent phenomenon in Pakistan where 97% of the population is Muslim, and religious as well as social norms strongly prohibit extramarital sex. The contours of this work have shifted, however, from geographically located brothel-based arrangements for sexual activity to a more dispersed configuration of sexual transactions carried out across the country’s cities. This has largely been the result of Islamic Hudood laws, introduced by the military government in 1979, and the subsequent years of the criminalization and penalization of the sex-worker community and the shutting down of several brothels and red-light areas by the state [25]. While the vast majority of street-based KSWs continue to survive in the cities through residing in “Deras” – strictly regimented, all-Khwaja-Sira residences, managed by a guru (group leader/network operator) that survive and function through the adoption of young Khwaja Siras abandoned by their families and communities [28, 29] – the places used for sexual transactions have moved considerably outside their jurisdiction.

During the last two decades, important shifts have occurred within commercial sex-work, which was historically organized around brothel-based patterns of sexual interaction. KSWs traditionally solicited clients through the guru and provided services at the dera. According to estimates from second-generation surveillance (SGS) system for HIV/AIDS, 34.7% KSWs were soliciting clients through gurus in 2005 [30]. Guru-based soliciting of clients from deras has decreased over the years to an estimated 22.7% in 2017. On the other hand, street-based soliciting of clients among KSWs since then has increased from 17.5% to 2007 to 33.3% in 2017 [9]. Street-based KSWs solicit their clients primarily in public places (e.g., parks, bus stops, traffic signals, commercial market areas) with limited or no mediation of the guru. Sexual services are provided at public places or a place chosen by the client usually away from the deras. In Pakistan, where links have been found between consistent condom use in KSWs and dependence on gurus, the increasing trend towards street-based sex work has important implications for HIV prevention [1]. However, the emerging needs and vulnerabilities of occupationally mobile street-based sex workers within changing interactional sexual contexts remain inadequately addressed.

## Methods

### Study site

The study was conducted between October 2017 and January 2018 with street-based KSWs in four neighborhoods of Lahore; Chaburji, Heera Mandi, Defense Housing Authority (DHA) and Johar Town. Lahore is the capital city of the biggest province Punjab in Pakistan and has the one of the highest presences of KSWs [9]. The study locations were chosen based on the known public presence of KSWs, which is in Heera Mandi (the red-light district in Lahore), and later through recommendations from already recruited respondents. Moreover, these neighborhoods represent different geographical areas of the city: Heera Mandi and Chaburji are lower-income neighborhoods are approximately 4.8 km apart in the old part of Lahore, one of it is associated with the historical red-light area of the city. DHA and Johar Town are located in the newly developed and relatively affluent sectors of the city, 36 km and 17.5 km away from the red-light district, respectively.

### Recruitment

Overall, 20 KSWs were recruited through purposive sampling. We partnered with a public health worker who had access to the KSW community and who was hired as a research assistant. KSWs were recruited by this research assistant through street recruitment in the selected four neighborhoods of Lahore. Inclusion criteria required that the participants (1) were at least 18 years old, (2) were self-reported Khwaja Siras, (3) were voluntary sex workers^1^ who solicited clients primarily through walking the streets, 3) had reported participating in anal intercourse during the previous 12 months, 5) had lived in Lahore for at least 12 months, and 6) were able to provide informed consent of their own free will. KSWs who completed the interview received PKR 1,000 ($7) compensation for their time which was not informed to them before the start of the interview. Provision of incentives to Khwaja Siras is a customary practice undertaken by researchers, private non-governmental organizations (NGOs) and media correspondents when seeking information from the Khwaja Sira community. This often results in conventional and normatively accepted responses from Khwaja Siras, because these are viewed as attempts at building public image of the community [26]. In order to build trust and rapport, we decided to inform the participants of incentives at the end of the interview.

### Data collection

In total, 20 in-depth interviews with KSWs which lasted approximately 60 to 90 min were conducted. The interviews were conducted by the researcher and her research assistant, in Urdu which is the local language, in locations chosen by the respondents. These locations included their homes or the homes of their friends, street corners, markets, and sidewalks. The semi-structured interview guide was originally designed in English and subsequently translated into Urdu. It consisted of two parts: The sociodemographic survey questionnaire included nine closed-ended questions about age, level of education, living arrangements, number of years in sex work, knowledge about spread of HIV, HIV status, risk perception and consistent usage of condoms. The question “How did you join this profession?” was used to screen sex workers who self-reported voluntary participation in sex work. Response options included: (a) voluntarily, (b) coercion by family members/guru (c) by fraud d) through abduction. The interview guide included open-ended questions about knowledge and use of condoms. Some examples of questions included “who do you use condoms with?’, ‘For which sexual activities are condoms used regularly?’, ‘What are the benefits of using condoms?’, ‘What are the challenges associated with using condoms regularly?’ The interview guide also included questions related to work environment, sexual activities and protective behavior, and characteristic of paying clients and non-paying partners. Some examples of the questions include, ‘how is street-based sex work different from brothel-based sex work?’, ‘‘what are the risks specific to street-based sex work’, ‘can you define the characteristics of your sexual partners?’, ‘Can you describe the sexual services provided to your clients?’, ‘how do you protect yourself during interactions with clients and other sexual partners?’. Finally, the interview guide had questions, ‘who do you seek for help for ensuring consistent use of condoms?’ and ‘who do you seek for help for your protection?’.

All KSWs who were literate provided informed written consent before the start of the interview. 10 respondents in the study had no education. The informed consent was read out to them by the researchers prior to beginning the interview. The interviews commenced after verbal consent was provided. All the interviews were audio-recorded, transcripts were translated into English and examined for accuracy after professional transcription. The researchers involved in data analysis were fluent in Urdu and English.

### Data analysis

The qualitative data was analyzed using six-phase process of reflexive thematic analysis [31–33]. We adopted a constructionist epistemology and an experiential orientation focusing on the meaning and meaningfulness as recognized by respondents in relation to condom using behaviors. The analysis process included familiarization with data through ‘active listening’ of recorded interviews and multiple readings of transcribed manuscripts to produce an initial set of codes during the first-level round. Both semantic and latent codes were generated predominantly inductively through open coding that underscored meanings of respondents. However, deductive analysis was done to ensure generation of meaningful themes with respect to the research question. This is in line with trends in qualitative analytical approaches which employ combination of inductive and deductive approaches simultaneously [33]. This was followed by a recursive “dual-level” review of themes with respect to both, data set and coded data items [31, 32]. This resulted in finalization of three themes during the final write up of the report; (1) Individual level factors influencing ICU among KSWs, (2) social and sexual network level factors influencing ICU among KSWs, and (3) community level influences on sexual practices and identities.

## Results

The sample characteristics are presented in Table 1. The initial coding of interview data confirmed three levels of risk factors for ICU among KSWs: (1) individual level factors, (2) social and sexual network level factors, and (3) community level factors. All themes and sub-themes are described in detail in the following sections.

**Table 1:** Sample characteristics of street-based KSWs in Lahore (n = 20)

Sociodemographic variables	n	%
**Age**		
18–25 years	6	30
26–35 years	8	40
36–45 years	5	25
46 years and above	1	5
**Education**		
No education	10	50
Primary level	7	35
Secondary level	3	15
**Living arrangement**		
With the guru	20	100
With family	0	0
**Number of years in sex work**		
Less than 5 years	8	40
More than 5 years	12	60
**HIV status**		
HIV positive	8	40
HIV negative	10	50
Unknown	2	10
**Knowledge that HIV/AIDS is transmitted through sex**		
Yes	20	100
No	0	0
**Consider yourself at risk of HIV/AIDS**		
Yes	14	70
No	6	30
**Consistent condom use**		
Yes	3	15
No	17	85

### Individual-level risk factors

#### Level of knowledge and awareness

The level of knowledge and awareness about HIV/AIDS, sexually transmitted infections (STIs) as well as safer sex practices among KSWs is critical in determining the consistency in condom use. Many respondents referred to HIV/AIDS as the “disease one gets from sharing bed”, referring to sexual intercourse.

Majority of the respondents in our study displayed awareness about HIV/AIDS, STIs and the necessity of condoms in protection against STIs.

“We know HIV is spread through sex if you don’t use condoms.” (30 years old KSW)

However, there were still important misconceptions that emerged as barriers to consistent condom utilization during sexual interactions. These misconceptions were more prevalent among KSWs of younger age. One major misconception among KSWs was that occasional non-usage of condoms did not pose a risk for contracting HIV or other STIs to those sex workers who were otherwise regular users of condoms. For instance, a 35-year-old KSW said:

“Many young KSWs believe that occasional sex without condoms is not harmful.” (35 years)

Another example of a common misconception is as follows:

“If you are regularly using condoms, occasional withdrawal method can protect you from HIV.” (23 years)

“Condoms are not necessary for oral sex.” (30 years)

#### Age

Among the respondents of this study, young age and perceived inexperience of KSWs between 18 and 25 years emerged as an important barrier to consistent condom use during sexual interactions with clients. Young KSWs mentioned that the negotiation process for condom utilization with clients was overwhelming, especially with clients who were older in age as compared to them:

“I am still new and learning. I don’t always have the confidence to convince the clients.” (20 years)

Two young sex workers believed that because they were less experienced as compared to other sex workers in the market, having sexual interaction with clients without condoms occasionally was a way of getting more experience and understanding of their work:

“I am young and I am learning. So, I sometimes don’t insist on condoms to see what difference does it make?” (18 years)

Younger KSWs are also preferred by clients and can seek higher prices for sex without condoms.

“If you are young, you can charge higher rate for sex without condoms.” (19 years)

One young respondent avoided condoms in order to deal with the lack of intimacy and feelings of alienation within street-based sex work. A 23-year-old respondent talking about young KSWs stated:

“They are young and easily heartbroken. They need love. So, when they don’t receive love, they don’t use condoms to feel love.” (23 years)

Sex work is a competitive market and some respondents shared that ageing in their profession was a major drawback in securing clients. Those above 40 years of age reported cases of ICU with clients as a result of declining client demand in sex work industry. Therefore, HIV risk was not always the most salient health concern among ageing sex workers. As a result of the demand for younger sex workers, all of the KSWs aged over 40 years reported being more compliant of their client’s demands with respect to condom use.

“They *[clients]* say, if we have to use a condom why not go to the younger one? And we comply because at the end of the day we have to eat.” (43 years)

“Now because I am older, I am not that attractive to many clients. I cannot risk losing clients because of demanding condoms.” (40 years)

One the other hand, most of the older KSWs also reported that street-based sex work was physically demanding. Four sex workers spoke, for instance, of sex work as an economic necessity resulting from discrimination within employment practices in the city. As a result of ageing, their mobility had become restrained and restricted within familiar neighborhoods and with regular clients with whom condom use declined:

“We’re too tired to negotiate. We stand or walk all day in this hot weather. We don’t have stamina for arguments now.” (38 years)

#### Pleasure and pain

Discussion of sexual pleasures and erotic desires is a taboo topic in Pakistan. However, sexual pleasure and erotic desires emerged as important considerations among KSWs in our discussions on condom use during sexual interactions. Condoms were believed to decrease the experience of pleasure. Experiencing pleasure emerged to be an important factor for younger KSW.

“I do not use condoms because I do not like them at all. If a person comes for anal sex then we use condoms. Otherwise, we do not *want* to. I don’t really *enjoy* using them either.” (23 years)

Sexual pleasure for these sex workers is complex and not solely restricted to anal penetration. However, anal penetration without condoms is a crucial element in the experience of pleasure and subsequently sexual identities:

“How can you be one of *us* if you haven’t experienced it as it is? […] They will not admit it but all of us have done it without it sometimes or most of the time. That’s how you find your *rooh* [soul/identity].” (30 years)

In addition to concerns related to pleasure, some respondents found the use of condoms by their partners during penetration as painful or uncomfortable. In such cases, condoms were not used to avoid experiencing anal pain:

“It hurts my back when they use condoms. I am sometimes afraid I will bleed.” (35 years).

#### Mental health issues/psychological factors

The majority of KSW respondents in this study pointed out the significance of their mental state in determining their choices regarding condom utilization.

“At the end of the day I feel very lonely and depressed. I have trouble sleeping at night. If I am feeling too low, I can get careless with clients.” (35 years)

Another KSW shared how thinking about or using of condoms itself produced anxiety related to acquiring HIV/AIDS and affected decision making regarding its utilization:

“Sometimes we get exhausted by worrying so much about using condoms and getting HIV/AIDs all the time. At the end of the day this is our only bread and butter. Nowadays I am so worried that I don’t even want to talk about it with anyone.” (25 years)

### Social and sexual network level risk factors

#### Perceived characteristics of sexual partners

In situations where KSWs were in a position to negotiate condom use, the decision to not use condoms in a sexual interaction with clients was sometimes based on misconceptions about HIV and STIs in general. One important misconception that was reported among interviewed KSWs was the belief that another person’s HIV status could be ascertained through their physical appearance or observed social status. As a result, all of the KSWs had developed their own methods of profiling suitors as potentially HIV positive or negative.

“Skin is the best reflection of someone’s health. If a client has suspicious spots, pimples or wounds or if their skin is burnt and dark, I know there is something wrong and at least I have to use condom. But sometimes at night if it is dark, I can’t observe properly.” (27 years)

People who were known to use drugs regularly were avoided as clients due to the prevalence of stigmatizing sentiments among KSWs.

“We have been doing this for so long, you can almost tell from the appearance if someone has HIV or any disease like that. These people who use drugs carry HIV around and it shows on their faces. The men who are healthy look clean.” (43 years)

Some also reported not using condoms with middle-class appearing men because they were assumed to be less risky. HIV was here labelled as a disease of either the wealthy people or individuals from low-income backgrounds, such as illustrated as follows:

“I look at their clothes to judge the status of clients. Middle class men are always in clean clothes. They also often prefer using condoms.” (43 years)

#### Dynamics of cruising spots and places of sexual interactions

The cruising spots and places of sexual interactions for KSWs in Lahore emerged as a key barrier to condom utilization. The cruising spots include traffic signals, bus stops or railway stations, commercial markets, and other accessible public venues. Engaging in transactional sex with a KSW is not only illegal but also considered disgraceful and downgrading for men in the heteronormative Pakistani society dominated by patriarchal values. For this reason, the exchange between KSW and clients is bound by time and the challenges of interacting in public spaces. The sex workers mostly receive resistance from clients on using condoms. Most respondents shared that successfully negotiating for condoms can be time-consuming and mostly discourages clients.

“Everything happens very fast in our business. We and our clients are all in a hurry for our own reasons. There is no time for thinking about or using a condom.” (35 years)

Longer public interactions between male clients and KSW in public places tend to attract attention of the police or the bystanders:

“If you insist on condoms too much sometimes the discussion can become heated. People here are just waiting for a show so they can gather around which is bad for clients.” (40 years)

Three respondents reported that the clients of KSWs are also vulnerable to physical, verbal and extortion by police or bystanders. As a result, the clients want to spend as little time at the cruising spot as possible.

“Clients are scared of the police especially if they are younger. They don’t want their family to find out and our scared of police beating. So, if you are too demanding or want to discuss matters like condoms they just move on quickly.” (27 years)

#### Competition in sex trade

KSWs mostly operate in groups of 4 to 5 or more at their designated cruising spots. This is a strategy to ensure safety and manage competition with other KSWs in the city. However, KSWs face competition from male and female sex workers in the vicinity. This competition negatively impacts condom negotiation and utilization between KSW and clients. Most of the respondents reported that they do not mention condoms on days when there are few clients or there are too many sex workers present at the same time.

“On weekends, you have more sex workers around here. So, we are all competing for the clients. Demanding men to use condoms on these days is difficult because they have more options.” (19 years)

#### Violence and lack of safety nets in street-based sex work

Cruising spots for KSWs and the places where sexual interaction takes place are often different. While the negotiations regarding price and condom utilization takes place at the cruising spot, the nature of sexual interaction is determined by the dynamics of the place where sexual interaction takes place. The payment is also made after the completion of the sexual exchange. These places as reported by KSWs ranged from public parks, motels, rented single rooms, private residences in suburban areas or nearby villages adjacent to the city, and cars. The majority of respondents shared that unlike brothel-based sex workers, KSWs working on the streets in most cases cannot decide the place where sexual interaction will take place. This provides complete control over the nature of sexual exchange and the decision regarding utilization of condoms to the clients.

“If I am with a group of clients, I feel more insecure and just comply with their demands. If they want to use condoms, I use them but if they don’t want to, I will just agree. Otherwise, they become violent and we cannot protect ourselves.” (30 years)

Many KSWs reported that exposure to violence, extortion and varying degree of sexual assault including forced sex for KSWs was common and affected the utilization of condoms.

“Once a man tied me to the tree after raping and beating me because I asked him to use a condom.” (40 years)

Lack of safety nets and provision of security makes them more compliant of the demands of their clients.

“No one comes to save us. Once these men raped me and left me unconscious in front of my house. My guru could do nothing. These men had taken me to their house and I was all by myself there. If these men don’t care about our dignity, why would they care to wear condoms. So, we just comply.” (35 years)

Many respondents reported frequent experiences of extreme physical and sexual violence as a result of lack of safety nets in unfamiliar places of sexual exchange. Such instances happened when the client belonged to politically influential families, was under the influence of drugs, or if there were multiple men present at the place of sexual exchange. In such situations, KSWs reported that condoms were least of their concern because they focused on getting out alive without injuries as quickly as possible:

“There is no use of discussing condoms when they are intoxicated. Once a client took me to the village. There were two or three men who were intoxicated and who sexually exploited me for two to three hours. When they were done with me, they gave me 500 rupees. I asked them for more but they didn’t give me. The man who picked me up, he accused me of stealing his phone. After that he started to beat me. There was no one to help me.” (27 years)

#### Condom use with lovers

Many Khwaja Siras maintain short-term and long-term, monogamous sexual relationships with heterosexual men referred to as *dost* (friend, lover) or *girya.* These male partners are equated to husbands with whom Khwaja Siras adopt the role of receptive sexual partners. Eight KSWs in the study mentioned that they did not regularly or occasionally use condoms with their *giryas* because they trusted and respected them and the nature of their relationship was different:

“I use condoms with my clients not him. He will not like it if I asked him to do it.” (30 years)

### Community level risk factors

#### Influence of guru and Dera culture

Experiences of marginality and violence originating from gender variance drives many gender non-normative persons into the Khwaja Sira communities in Pakistan. These communities provide structured care within strictly regimented, hierarchical peer networks. These peer networks are regulated through the traditional *Guru-chela* (master-apprentice) *relationship.* Many Khwaja Siras live in conditions of poverty after being abandoned by their families and joining Khwaja Sira communities. The young novices are trained and disciplined into Khwaja Sira livelihood and lifestyle under the guidance of their guru who acts both as a parent and a mentor [5].

Even for Khwaja Siras who work on the streets, the gurus are important in providing a relatively safe residence, a cultural identity and social status in the Khwaja Sira community. For this reason, gurus exert immense influence on their chelas’ sexual practices inside and outside the deras. According to our study’s findings. the gurus played a critical role in fostering safe or risky sexual behaviors among street-based sex workers as well.

KSWs reported that ensuring sexual health and safety of chelas during sex work was not a priority of many gurus. Some of them were found to enforce their will on the chelas through financial penalties, physical and verbal abuse, and imposition of social boycott:

“I work in this area; our guru doesn’t allow us to go anywhere else. He doesn’t care about our protection. He hurts us, physically abuses us, and calls us names. If we don’t share our money with him, he will cut our hair, tear our clothes and blacken our faces. He will even kick us out. Do you think he cares if we use condoms?” (23 years)

Particular communal penalties enforced by gurus were found to negatively influence condom use behavior among the chelas during street-based sex work. For instance, some respondents said that gurus often imposed penalties on their chelas as a result of some violation:

“My previous guru was very strict. If I ever disagreed with him, he would confiscate all my belongings. Once, I said that at least give me my condoms before I leave for work. But you could never change his mind.” (35 years)

Only chelas of gurus are allowed to engage in traditional Khwaja Sira sources of income within their designated areas. The majority of KSWs spoke how violation of community codes of conduct could increase their risks for ICU. For instance, some KSWs mentioned that soliciting clients in the designated area of another guru often entailed episodes of violence and abuse from other KSWs:

“If I go to another guru’s area and if other KSWs catch us, they beat us up, cut our hair, take our belongings and throwaway our condoms. Whatever they want, they do.” (25 years)

In addition, gurus act as protectors of their chelas and are responsible for their conduct within the community and the city at large. Most Khwaja Sira communities manage societal stigma and avoid police harassment by maintaining an image of piety, retaining ambiguity about their corporeality, hiding their sexualized lifestyles, and publicly engaging in begging only. A threat to this public image is seen as a threat to the survival of the community. In this situation, KSWs reported that condoms utilization is an important threat to this collective image and makes it difficult for them to buy condoms from shops in their neighborhoods. Purchasing of condoms is discouraged by the gurus who also become a barrier to accessing condoms provided by NGOs:

“My guru is a kind soul and doesn’t believe in sin [implying sex]. The social workers come to our houses with condoms and our guru throws them away so the police will not bother us, or those kinds of men would not find their way to our *dera*.” (35 years)

#### Changing urban geography of sex work

Street-based KSWs’ sexual and social networks are larger but relatively more dispersed as compared to those who engage in commercial sexual activities through brothels or deras. One drawback of increasing reliance on street-based sex work among KSWs is the loss of traditionally available safety nets within brothel-based sex work. For instance, two KSWs shared that there was comparatively more room for condom negotiation and less fear of violence from clients when sex workers were operating from brothels because of presence of pimps, local police officials with whom KSWs had established informal links, and the support of their own community or local community-based organizations (CBOs).

“When I was working from my house, I could threaten the client that I would call my people if they didn’t wear condoms or if they became aggressive. On the streets we are just on our won.” (46 years)

According to respondents, the gentrification of the traditional red-light district has consequently increased the presence of urban tourists, vendors and law enforcing agents in the neighborhood. As a result, brothel-based sex workers are finding their work being forced onto the streets away from the red-light district. This is one of the reasons why most of the KSWs in this study were compelled to work on the streets:

“Now there are bus tours and walking tours around the red-light district. This place is not very workable for sex workers. We prefer to walk for hours to our spot where clients would be comfortable approaching us and there is less presence of police. But I prefer not to carry condoms because we have to pass police check posts and they often tease us.” (40 years)

Gentrification has not only pushed KSWs onto the streets but also changed the clientage of sex workers operating on streets.

“We are told that men from good families would come to our doors. Now on the streets we are approached by clients who are *ghareeb* (poor). They hardly have any money or condoms. They are just interested in cheap and quick sex.” (27 years)

#### Discrimination, harassment and regular evictions

The closure and policing of the historic red-light area in Lahore has forced many KSWs to relocate in adjacent working-class neighborhoods in the city. However, over the years, KSWs have faced discrimination and regular evictions from their homes because of prejudice within larger society against their traditional practices. Therefore, many KSWs build *izzat* (respectability) by begging in morning and refraining from engaging in sex work within their neighborhoods. They engage in sex work mostly at night and through street-based soliciting of clients from cruising spots away from their places of residence. This adds costs related to transport, increases chances of exposure to violence and regular physical, verbal and monetary harassment by police officers. Most KSWs added that they avoided carrying condoms because of the fear of police harassment:

“Every day we are stopped at police check-posts and we pay them to let us go so we can earn some money.” (20 years)

Another KSW mentioned the regular harassment they faced at hands of the law enforcing agents:

“They often take us to the police station; especially if they find condoms and keep us there for a night or until my guru comes to get me.” (30 years)

#### Networks with NGOs and CBOs

Most of the respondents shared that they face harassment by shopkeepers if they directly try to buy condoms from the shop. Many respondents also added that they could not afford to buy condoms all the time and as a result relied on local healthcare workers, non-governmental organizations (NGOs) or CBOs for accessing condoms without a charge or at subsidized prices. However, there were a number of challenges that were reported in accessing free condoms.

The public sector in Pakistan lacks the capacity to extend healthcare related services directly to high-risk groups. This has resultantly increased the dependence of the state on NGOs for provision of HIV prevention services. Many local NGOs and CBOs hire or collaborate with KSWs to forge links with and develop trust within the KSW community. While this has proven to be an effective strategy, personal rifts between gurus or their *chelas* poses some serious challenges to the provision of condoms and other services [25]. According to the findings of this study, respondents perceived Khwaja Siras working for NGOs as holding stigmatizing attitudes towards KSWs. KSWs also accused most of the NGO workers of being corrupt and allies of the West. As a result, five KSWs reported avoiding engaging with Khwaja Siras working with NGOs or disclosing their personal and professional information to them:

“These NGO workers come and throw condoms in our houses from outside. They have no respect for us. So, we throw their condoms back at them.” (27 years)

In addition, many KSWs found NGO workers as intrusive, insensitive and unreliable:

“The NGO is corrupt. They only take care of their own people, the khwaja saras who work for them. But not us. We went for a checkup of our HIV status and for some condoms, but the females that worked there asked very private questions: How much sex do you have? How much does the penis go in? They were such personal questions that even we were embarrassed to answer them. Still, they did not give me the results or condoms. It is totally corrupt… You see they earn from it. They get more money if they can interview more transgender people.” (39 years)

Furthermore, HIV prevention and treatment endeavors have slowed down in Pakistan as a result of unsteady policies and dwindling donations from donor agencies. Many KSWs complained that there was an absence of stable prevention efforts in their localities including provision of free condoms and lubricants among KSWs:

“Sometimes they have condoms, and at other times we have to manage on our own.” (23 years)

“These Khwaja Siras in NGOs think they are better than us and look down on us. When they give us condoms, they tell us to choose the right path and leave sex work. I don’t interact with these NGOs at all.” (20 years)

While HIV prevention services are important facilitators of condom use, street-level implementation of these policies can become barriers to IUC as a result of stigmatizing attitudes held against targeted communities.

## Discussion

The results of this study highlight multi-level risk factors for ICU among street-based KSWs. A key finding of this study is that multiple barriers exist that make sustained use of condoms during sexual interactions difficult for KSWs. The responsibility for condom use in street-based sex work lies s largely with the individual sex worker who lacks institutional support, provision of safety nets and/or regular incentives by the government for using condoms. The findings are important as recent research in Pakistan has revealed a remarkably low degree of knowledge about condoms and awareness of their proper and accurate use among MSMs and transgender women [8].

Importantly, our research demonstrates that the availability of financial incentives is an important motivator for KSWs to choose not to use condoms during sexual interactions with clients. This is in line with studies that have found that financial instability of sex workers is inversely linked with their capacity to negotiate for condom use with their sexual partners [34]. The likelihood for engaging in risky sexual practices is higher for sex workers living in poverty [35, 36], including lack of condom use [37]. This is important in context of street-based KSWs as street-based sex workers have lower earnings than sex workers who work in indoor venues including bars [37] and brothels [38], and many KS in Pakistan have unpaid debts [39]. Therefore, condom-related interventions with sex worker communities in Pakistan should be centered on improving and sustaining financial security of sex worker communities. For example, Bill and Melinda Gates Foundation’s India AIDS Initiative (*Avahan*) in Karnataka, South India, was able to increase and maintain consistent condom use behavior in sex worker populations by addressing their financial vulnerabilities. The program achieved this by increasing financial literacy of sex workers, assisting them in opening savings accounts and providing guidance on financial investments [40]. In Pakistan, where KSWs live in communal groups, improving financial security of individual sex workers will also lead to collective empowerment of a marginalized community.

Health policy in Pakistan tends to direct HIV-related interventions at the individual level of risk behaviors [9], but has overlooked key occupational differences within risk populations. Our study suggests that street-based KSWs have specific occupational risk factors for ICU. The study participants described street-based sex work as a dangerous and uncertain occupation, because multiple risks associated with places of sexual exchange and clients could not be ascertained and managed beforehand. This uncertainty was seen as an outcome of their occupational mobility and lack of safety nets in sex-work venues. Similar findings have been reported by a study conducted among female sex workers in India which has found an association between high HIV risk and small-scale mobility of sex workers [41]. In addition, police repression was highlighted as a primary concern among respondents. In criminalized settings around the world and in Pakistan, street-based sex workers have been found to develop antagonistic relationships with police that limits their ability to report abuse without their safety and economic security being compromised [42, 43]. In addition, police violence has been found to increase sex workers’ vulnerability to client violence and STIs by disrupting their informal networks and pushing them to less visible places where sexual services are exchanged. [44]. While decriminalizing sex work in a Muslim majority country might be challenging, police violence and exploitation of sex workers must be addressed for increasing effectiveness of HIV prevention programs for sex workers in Pakistan. Interventions that combine community solidarity with government policy actions have been found to successfully increase uptake of condoms among sex workers and reducing STIs in India, Puerto Plata and Santo Domingo [45, 46]. For example, Bill and Melinda Gates Foundation’s India AIDS Initiative (*Avahan*) in Karnataka, South India successfully reduced police violence through advocating and sensitizing police officials along with empowerment of FSW groups to deal with police violence [45].

In addition, street-based KSWs are not a homogenous group and our respondents displayed a range of individual behaviors and reasons for forgoing condom use. For instance, discomfort and pain as a result of condom use during receptive sex was identified as a key barrier among respondents of this study. Use of condom compatible lubricants CCLs [47] and increasing access to a wide range of condoms in terms of size and texture have been found to improve uptake of condoms [48]. National Aids Control Program (NACP) in Pakistan provides at-risk groups with condoms and lubricants. However, more evidence-based research is required on knowledge, practices and accessibility of condoms and lubricants in sex worker communities in Pakistan. In addition, continued counseling and education are needed to encourage uptake of condoms and lubricants.

Importantly, some respondents acknowledged that ICU was linked with the need for seeking pleasure during sex. Unfortunately, pleasure is a stigmatized and taboo topic in healthcare settings and interventions in Pakistan. Our findings suggest that a sex positive approach [49] needs to be incorporated within the HIV intervention programs in Pakistan in order to achieve safer sexual experiences for sex workers and their sexual partners. The current condom promoting interventions in Pakistan are under HIV prevention programs of NACP which predominantly operates on risk-based models. However, condom use interventions centered on promotion of sexual pleasure have been found to be successful in increasing uptake of condom without reinforcing fear or shame in Australia, Mozambique and Cambodia [50]. In addition, we suggest that healthcare providers and social workers should be provided culturally-sensitive trainings in how to address issues of sexual pleasure in non-stigmatizing ways.

In our study, the majority of the KSWs were unsuccessful in consistently using condoms with their sexual partners. Our findings indicate the need for policy changes at macro and micro-level e.g. increasing availability of variety of condoms and lubricants at cruising spots and in the community, addressing the influence of economics on a KSW’s capacity to negotiate condom use, adopting a sex positive approach in HIV intervention programs and healthcare settings and addressing violence from street level bureaucrats. In addition, awareness of human rights of KSWs, creations of linkages of KSWs to community-based legal services, social entitlements and job opportunities are needed to uplift their social status in Pakistan [51].

### Limitations

The present study has a number of limitations. Firstly, the findings are derived from self-reported data through in-depth interviews with street-based KSWs. Our estimates of high-risk and protective behaviors are prone to social-response-related reporting bias and, therefore, may be underestimates or overestimates, respectively. Lastly, the data was collected during 2017–2018 before COVID-19 and as a result does not include information about how ICU is impacted by the pandemic, subsequent nationwide lockdowns and launching of Pre-Exposure Prophylaxis Program (PrEP) in Pakistan. PrEP was introduced in Karachi on June 7, 2022, by the Health Department of the Government of Sindh in cooperation with the United Nations in Pakistan and currently there are no studies on PrEP and its impact on condom use among sex workers from Pakistan [52].

## Conclusion

In this paper, we have adopted a socio-ecological framework to investigate ICU within the risk contexts for HIV transmission among transgender street-based sex worker in Lahore, Pakistan. The results of this study are exploratory in nature but offer nuanced and first-hand evidence for condom utilization patterns of at-risk, street-based KSWs in Lahore. In doing so, our findings support the central role that HIV risk contexts play in exposing KSWs to the risk of contracting HIV by creating barriers to the access and utilization of condoms during commercial and non-commercial sexual interactions.

## Data Availability

The qualitative datasets generated and/or analysed during the current study are not publicly available due to the data containing information that could compromise research participant privacy/consent but are available from the corresponding author on reasonable request.

## Footnotes

1 Voluntary sex workers were screened by the following question in the sociodemographic section of the survey: “How did you join this profession?” Response options included: (a) voluntarily, (b) coercion by family members/guru (c) by fraud, or (d) through abduction.

## List of abbreviations

AIDS: Acquired immunodeficiency syndrome
CBO: Community-based organizations
HIV: Human immunodeficiency virus
ICU: Inconsistent utilization of condoms
KSW: Khwaja Sira sex workers
MSEM: Modified Social Ecological Model
NGO: Non-governmental organization
